# Correcting biases in psychiatric diagnostic practice in Northwest Russia: Comparing the impact of a general educational program and a specific diagnostic training program

**DOI:** 10.1186/1472-6920-8-15

**Published:** 2008-04-04

**Authors:** Grigory Rezvyy, Alexander Parniakov, Elena Fedulova, Reidun Olstad

**Affiliations:** 1 Nordland Hospital, Psychiatric Department, N-8092, Bodø, Norway; 2 Northern State Medical University, 51 Troitsky avenue, 163061, Archangelsk, Russia; 3 Archangelsk County Psychiatric Dispensary, 262 Lomonosova avenue, 163061, Archangelsk, Russia; 4 University Hospital in Northern Norway, Åsgård, 9291, Tromsø, Norway; 5 University of Tromsø, Faculty of medicine, Institute of clinical psychiatry, Åsgård, 9291, Tromsø, Norway

## Abstract

**Background:**

A general education in psychiatry does not necessary lead to good diagnostic skills. Specific training programs in diagnostic coding are established to facilitate implementation of ICD-10 coding practices. However, studies comparing the impact of these two different educational approaches on diagnostic skills are lacking. The aim of the current study was to find out if a specific training program in diagnostic coding improves the diagnostic skills better than a general education program, and if a national bias in diagnostic patterns can be minimised by a specific training in diagnostic coding.

**Methods:**

A pre post design study with two groups was carried in the county of Archangels, Russia. The control group (39 psychiatrists) took the required course (general educational program), while the intervention group (45 psychiatrists) were given a specific training in diagnostic coding. Their diagnostic skills before and after education were assessed using 12 written case-vignettes selected from the entire spectrum of psychiatric disorders.

**Results:**

There was a significant improvement in diagnostic skills in both the intervention group and the control group. However, the intervention group improved significantly more than did the control group. The national bias was partly corrected in the intervention group but not to the same degree in the control group. When analyzing both groups together, among the background factors only the current working place impacted the outcome of the intervention.

**Conclusion:**

Establishing an internationally accepted diagnosis seems to be a special skill that requires specific training and needs to be an explicit part of the professional educational activities of psychiatrists. It does not appear that that skill is honed without specific training. The issue of national diagnostic biases should be taken into account in comparative cross-cultural studies of almost any character. The mechanisms of such biases are complex and need further consideration in future research. Future research should also address the question as to whether the observed improvement in diagnostic skills after specific training actually leads to changes in routine diagnostic practice.

## Background

"Diagnostic assessment is a fundamental aspect of clinical care. It involves gathering key information in order to describe and understand the patient's clinical condition" [1] and forms the basis for effective treatment plans. The diagnostic practice should generally be compatible with current international standards and "be as highly reliable and valid as possible" [1].

The ICD-10 classification system is "an international consensus of what at present is considered the most useful and correct way of classifying mental and behavioural disorders," [2] and has been used as the official diagnostic classification in many countries of the world over the past two decades [3,4]. Development of cross-culturally applicable diagnostic criteria and instruments for assessment of mental disorders in different cultures has been one of the major goals of the activity of the World Health Organisation (WHO). This reflects the commitment of the WHO to the development of a "common language" that will allow mental health professionals in different countries to understand one another and work together [5,6].

Despite of increasing use of the ICD-10 classification system in the most of the European countries, there are still differences in the use of the current classification system in cultures with different diagnostic traditions. Diagnostic practice in psychiatry seems to be very sensitive to cultural, political and other differences between the countries [7-9]. In an earlier comparative study of psychiatric diagnostic practice in Northern Russia and Northern Norway with case vignettes [10], the Russian clinicians used a greater variety of diagnoses than their Norwegian colleagues in those cases with neurotic, stress-related and affective disorders, as well as for the case representing an emotionally unstable personality disorder. In addition, the Russian psychiatrists tended to use schizophrenia and schizophrenia-like diagnoses when cases presented with psychotic symptoms. Among Russian psychiatrists somatoform disorder was a more common diagnosis in the case with agoraphobia. The Norwegians often weighted the affective aspects more than the psychotic symptoms in the case of schizoaffective disorder and tended to overestimate the degree of depression [10]. Such so called "national diagnostic biases" [11] may be traced to different historical, diagnostic and educational traditions in different countries. Also this national variation might result from possibly somewhat unclear formulations in the current diagnostic system. This could allow for cultural differences both in the definitions and in interpretations of the different diagnostic concepts.

The need to further develop the quality of psychiatric diagnostic assessment across the world has led the World Psychiatric Association (WPA) to instigate the development of the International Guidelines for Diagnostic Assessment (IGDA) in the mid 1990s [1]. Through this effort the WPA was making an innovative contribution to a higher standard in international diagnostic practice. The WPA educational program in diagnostic practice has been a very useful tool for the medical education of psychiatrists around the world. The program has been developed from a multicultural perspective, translated into many languages and is easily accessed through the Internet. The ICD-10 Training Kit, a program developed by the WPA in collaboration with WHO, is also available on WPA Online [12]. However, despite of the availability of various clinical guidelines and educational programs developed for the purpose of improving diagnostic practice in psychiatry, these programs need to become part of an existing undergraduate/postgraduate educational curriculum for psychiatrists. In some countries, there is a once-only educational process that leads to a formal authorization as a specialist in psychiatry. In this situation it would seem necessary to utilize postgraduate educational programs to improve diagnostic practice. In other countries, there are mandatory systems of continuing education, and it could be possible to incorporate systematic diagnostic training into an existing program.

Many researchers have noted a significant impact of education/training programs on the knowledge, the recognition and treatment of psychiatric disorders. However, there have been few reports evaluating the effect of the training programs in the use of current international diagnosing classifications [11,13]. Levav et al. [14] registered that the WPA general training program for primary care physicians has improved their knowledge about depression but "there was no evidence that the diagnosis of depression was made more frequently" after the program was completed. This would seem to indicate that a general education about a disorder does not necessary lead to a change in diagnostic practice.

In our experience, a major part of the postgraduate medical curriculum is traditionally dedicated to the teaching of the art and science of diagnosis in a general way. The classification system is often used as additional teaching material with participants being just informed about it. At the same time, some publications report about specific training in diagnostic coding [11,15] that is established "to facilitate implementation of ICD-10 coding practices". However, we did not find reports of studies comparing the impact of these two different approaches on diagnostic practice.

Our hypothesis was that establishing a diagnosis according to ICD-10 criteria is a specific skill, and that specialised training is necessary to improve this skill. Thus, a general education in psychiatry without this specialised training will not lead to proficiency in diagnostic skills.

The aim of the current study is to answer on the following questions:

1. Does a specific training program in diagnostic coding improve the diagnostic skills better than a general education program?

2. Can the previously described biases in North Western Russian diagnostic patterns be minimised by a specific training in diagnostic coding?

3. Do background factors such as age, gender or professional experience influence the ability to improve diagnostic skills?

## Methods

The current study was carried out between spring 2004 and autumn 2006 in the county of Archangels, Russia. A pre post design, utilizing two groups, an intervention and a control group, was employed.

### Educational programs

In the Archangelsk County, every specialist in psychiatry has to re-establish his/her professional qualifications every 5th year by attending a course and passing a clinical test. The education program runs continuously in small groups of 10–20 participants.

The original course is largely theoretical, 2 months in duration, and consists of several parts: diagnostic methods (medical, psychological, functional etc) in psychiatry (24 hours), main psychiatric syndromes (50 hours), psychiatric disorders (60 hours), treatment and rehabilitation of psychiatric patients (40 hours), some issues in neurology and clinical psychology (48 hours), psychiatric service organization (12 hours) and other relevant issues (this is the General educational program, the Control group in the current study).

As part of the research project, a specific training program in diagnostic coding (ICD-10 clinical guidelines for diagnosis) was implemented in the 2-month general educational program for every other educational group. The training program was based on the ICD-10 Training Kit. Case-vignettes from the ICD-10 Training Kit were translated to Russian and used as clinical illustrations in diagnostic workshops. The program lasted 5 days. Each day a separate workshop focused on a specific diagnostic group (organic and alcohol-related disorders, schizophrenia and other psychotic disorders, affective disorders, neurotic and stress-related disorders and personality disorders). The main activity or focus of the workshops was the evaluation of clinical written case-vignettes illustrating the actual topic. Every workshop lasted 5 hours and consisted of several parts: evaluation of the respective case-vignette by every participant independently, common discussion of the results, relevant clinical information, discussion and evaluation of the actual cases (Specific training in diagnostic coding, the Intervention group in the current study). The total length of the education program was not changed, but the other parts of the program were compressed in order to give space for the specific diagnostic training.

### Participants

Alternating groups of psychiatrists taking the required refresher course were given the specific intervention (the training program in diagnostic coding) as part of the refresher (total N = 45), while the control group (total N = 39) consisted of those who underwent the same general education program without the specific training in diagnostic coding. Together the 84 psychiatrist participants represented 55% of the psychiatrists working in Archangelsk County.

None of the participants in the present study had participated in a previous study of diagnostic practices [10].

### Assessment of diagnostic skills

#### Pre test

The first assessment was carried out on the first day of the course for both the control and intervention groups. All participants read the 12 written case-vignettes that were taken from a casebook developed for the introduction of the ICD-10 diagnostic system [16]. They then made their independent diagnostic evaluations. The same case-vignettes had been used in the previous study [10]. The case vignettes had been edited and reviewed by several ICD-10 training centre directors and selected as typical of the diagnostic categories they represented. These clinical examples were selected from the entire spectrum of psychiatric disorders and considered representative of daily practice. Every case vignette had been given a "right answer" – the diagnosis suggested by the experts. The doctors were asked to provide one diagnosis and possible additional diagnoses in every case. The first diagnosis should be taken as the primary and most preferred one. In the present study, only the first diagnosis is used in the analysis. After the first assessment, the case-vignettes were collected and the education program begun. The results from the first test were not shared with the participants and discussions about the results were discouraged. The participants were not informed about a subsequent second assessment at the completion of the educational program.

#### Post test

The second assessment was carried out during the last week of the course, approximately 7 weeks after the first test. All participants evaluated the same 12 case-vignettes with the instruction to evaluate these cases again on the background of the knowledge they had gained during the course. After the second assessment, the case-vignettes were collected and the results discussed.

### Statistical analyses

Data were coded and analysed using SPSS for Windows software (version 11.0). A correct diagnosis for a case was given a score of 1 and summed to a sumscore ranging from 0 to 12 for each participant. The sumscore difference between the intervention group and control group before and after education was evaluated using independent group non parametric tests (Mann-Whitney test); while changes of sumscore before/after education in each group were evaluated using Wilcoxon Signed Ranks Test. Differences between the numbers of correct versus wrong diagnosis for each case was tested (Pearson Chi-square test). P level < 0.05 was considered to be significant.

To explore the relationship between diagnostic improvement (pre post difference) and the background factors which could influence the results, a linear regression analysis, including both groups, was performed. The normally distributed pre post difference of the number of correct diagnoses was the dependent variable. Potentially contributing factors such as working place, experience with different patients, gender, age, years of medical experience or experience as a specialist were used as independent variables. Categorical variables such as working place were recoded into dummy variables. Experience with different patients was dichotomised (working with serious mental disorders/not working with serious mental disorders) before entering the analyses.

The regional ethical committee for research considered it unnecessary to evaluate the project due to the fact that the project included healthy persons and that non sensitive information was collected.

## Results

### Comparability of the study groups

Relevant information about the participants background and experience (gender, age, years since medical training, number of years as a specialist in psychiatry, number of years working in psychiatry, the type of patients they largely worked with; severe mental illness, addiction, neurosis, gerontopsychiatry, other), and their current work setting (hospital, community mental health centres (CMHC), district psychiatric offices and other) was collected.

The two participant groups were comparable in gender, age, experience as a physician and work setting (Table 1).

**Table 1:** Description of the participants

	**Intervention group (N = 45)**	**Control group (N = 39)**	**Total (N = 84)**
**Sex **Male:female ratio	0,73	0,95	0,83
**Age **(Mean ± SD)	45,6 ± 11,9	42,5 ± 12,0	44,2 ± 12,0
**Experience as specialist**			
Years as medical doctor (Mean ± SD)	20,0 ± 10,8	17,0 ± 11,7	18,6 ± 11,3
Years as specialist (Mean ± SD)	15,4 ± 9,6	14,7 ± 10,0	15,0 ± 9,8
Worked more than 4 years in psychiatry N (%)	42 (93, 3%)	36 (92, 3%)	78 (92, 9%)
Worked more than 15 years in psychiatry N (%)	21 (46, 7%)	17 (43, 6%)	38 (45, 2%)
**Main experience**	Number of persons/percent	Number of persons/percent	Number of persons/percent
Severe mental illness	23 (51, 1%)	16 (41, 0%)	39 (46, 4%)
Addiction	2 (4, 4%)	3 (7, 7%)	5 (6, 0%)
Neurosis	3 (6, 7%)	2 (5, 1%)	5 (6, 0%)
Gerontopsychiatry	4 (8, 9%)	1 (2, 6%)	5 (6, 0%)
Other	13 (28, 9%)	17 (43, 6%)	30 (35, 6%)
**Current work place**	Number of persons/percent	Number of persons/percent	Number of persons/percent
Hospital	19 (42, 2%)	16 (41, 0%)	35 (41, 7%)
Community mental health centers	14 (31, 1%)	12 (30, 8%)	26 (31, 0%)
District psychiatrists	5 (11, 1%)	3 (7, 7%)	8 (9, 5%)
Other	7 (15, 6%)	8 (20, 5%)	15 (13, 2%)

### Does a specific training program in diagnostic coding improve the diagnostic skills better than a general education program?

There was no difference in the sumscores between the control group and the intervention group at the first assessment (not shown). As shown in Table 2, at the assessment after education, both groups showed significant improvement in the skills to use the diagnostic classification system. However, results in the intervention group were significantly better than in the control group.

**Table 2:** Correct responses (%) and sumscores for the primary psychiatric diagnosis before (T1) and after (T2) a specific educational program in diagnostic coding (intervention group) compared with responses by psychiatrists before (T1) and after (T2) a general educational program (control group)

Case	Intervention group N = 45		Control group N = 39				
	Before (%)	After (%)	Before (%)	After (%)	^1^p-value	^2^p-value	^3^p-value
Dementia in Alzheimers disease (F 00.0)	67	93	49	80			
Alcohol withdrawal state with delirium (F 10.4)	73	96	74	72	**	**	
Acute polymorph psychotic disorder without symptoms of schizophrenia (F 23.0)	7	36	5	18		**	
Schizoaffective disorder (F 25.2)	29	89	21	36	***	***	
Bipolar affective disorder, manic with psychotic symptoms (F 31.2)	16	73	33	41	**	***	
Moderate depressive episode (F 32.1)	36	44	41	41			
Agoraphobia, with panic disorder (F 40.0)	4	60	3	13	***	***	
Social phobia (F 40.1)	56	82	39	56	*	*	
Obsessive – compulsive disorder (F 42.1)	7	33	8	13		**	
Post – traumatic stress disorder (F 43.1)	82	87	87	87			
Schizoid personality disorder (F 60.1)	84	98	85	97			
Emotionally unstable personality disorder. Borderline (F 60.3)	47	87	56	62	*	***	
**Sumscore**	5.11 ± 2.0	8.69 ± 2.3	5.05 ± 1.8	6.15 ± 1.9	***	***	**

^1^p-value when intervention group is compared with control group at T 2 (chi-square test (Pearson) for individual cases, Mann -Whitney Test when comparing sumscores)

^2 ^p-value when test results at T 1 are compared with T 2 in the intervention group (chi-square test (Pearson) for individual cases, Wilcoxon Signed Ranks Test when comparing sumscores)

^3 ^p-value when test results at T 1 are compared with T 2 in the control group (chi-square test (Pearson) for individual cases, Wilcoxon Signed Ranks Test when comparing sum scores)

*P < .05, **P < .01 ***P < .001.

Despite of the equal sumscores before education, minor differences in the diagnostic patterns were detected between the groups. Before education, participants in the control group were more accurate in diagnosing of bipolar affective disorder, while the intervention group showed better results in diagnosing dementia in Alzheimer's disease and social phobia. However, there were no significant differences between the groups on any case vignette before education when each case was analyzed separately. In general, there was considerable variation in the percentage of correct answers from the cases that received the most correct answers (schizoid personality disorder, alcohol withdrawal state with delirium, post-traumatic stress disorders) and the cases that received the least number of correct answers (agoraphobia, obsessive-compulsive disorder, acute polymorph psychosis). These variations existed in both groups. However, improvement was generally greater in the intervention group.

### Can the previously described biases in North Western Russian diagnostic patterns be minimised by a specific training in diagnostic coding?

In order to demonstrate how the specific training in diagnostic coding compared to a general educational program influence the previously described national biases, and to show distribution of the "not correct diagnosis", we have chosen to illustrate the most interesting cases in more details:

#### Schizoaffective disorder (F 25.2) (Figure 1)

**Figure 1:**
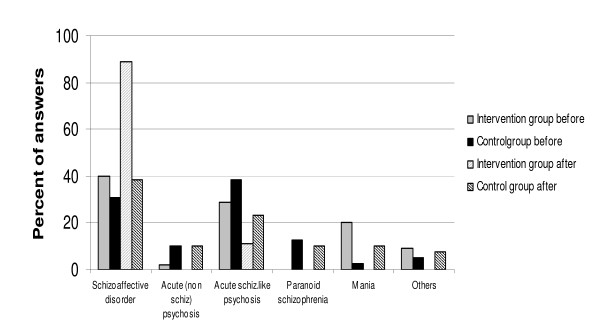
Differences in diagnostic pattern in the control group and the intervention group before and after the training program – Schizoaffective disorder case.

Initially most participants considered the case to represent diagnosis either F 25.2 (Schizoaffective disorders) or F 23.1 (Acute polymorphic psychotic disorder with symptoms of schizophrenia) although twenty percent of those in the intervention group chose mania with psychotic symptoms (F 30.2 and F 31.2). At post test the schizoaffective disorder diagnosis increased to 89 % in the intervention group, while the increase in the control group was not as dramatic and largely resulted from fewer F23.1 diagnoses being made.

#### Acute polymorphic psychotic disorder without symptoms of schizophrenia (F 23.0) (Figure 2)

**Figure 2:**
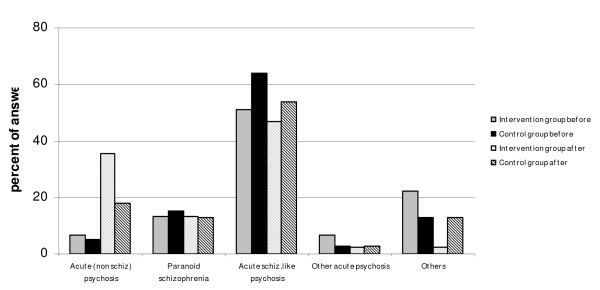
Differences in diagnostic pattern in the control group and the intervention group before and after the training program – Acute poly morph psychotic disorder without symptoms of schizophrenia.

Participants in both groups usually chose the correct main diagnostic category, F 23, but preferred code F 23.1 (Acute polymorph psychotic disorder with symptoms of schizophrenia) and F 23.2 (Acute schizophrenia-like psychotic disorder). Diagnosis F 20.0 (paranoid schizophrenia) was also chosen more often than the correct one. Despite of the considerable increase in the number of correct diagnoses after education (statistically significant change in the intervention group), the number of diagnoses with codes F 23.1, 23.2 and 20.0 were not considerably decreased. Still 60% of the participants in the intervention group suggested schizophrenia and schizophrenia-like diagnoses, while the number of correct diagnoses increased from 6.7% to 35.6% while the number of other diagnoses decreased dramatically (F 25 – from 11 % to almost 0 %). Although the control group improved somewhat the change was not significant.

#### Agoraphobia, with panic disorder (F 40.0) (Figure 3)

**Figure 3:**
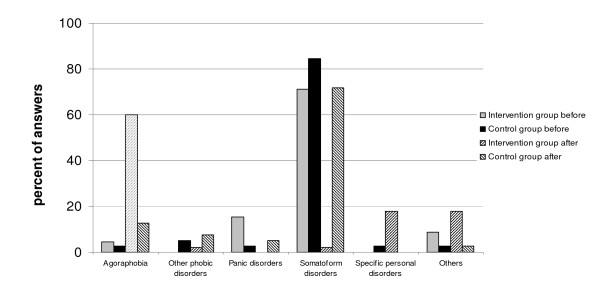
Differences in diagnostic pattern in the control group and the intervention group before and after the training program – Agoraphobia, with panic disorder.

There were only a few participants in both groups who chose the correct diagnosis at the first assessment, with most participants considering the case an example of a somatoform disorder. This changed dramatically at post test but only in the intervention group: the diagnosis of somatoform disorder had almost disappeared with 60% choosing agoraphobia and about 20% – personality disorders (F 60.5 and F 60.6). In the control group, there was some increase in the number of the correct diagnoses and some decrease in the use of the somatoform disorder diagnosis.

### Do background factors such as age, gender or professional experience influence the ability to improve diagnostic skills?

Linear regression was used to analyse the results after education in both groups of clinicians. Differences in sum scores were predicted by the potentially contributing factors such as work setting, experience with different patient categories, gender, age, years of medical practice or experience as specialist. Only belonging to the intervention group or control group (B = -2.472; SE = 0.45; P < 0.001) and working at a community mental health centre (B = -0.985; SE = 0.48; p < 0.05) predicted improvement. These two factors explained 30% of the variation in sumscores (data not shown). Belonging to the intervention group or control group alone explained 27% of the variation in sumscores (B = -2.354; SE = 0.46; P < 0.001).

**Figure 4:**
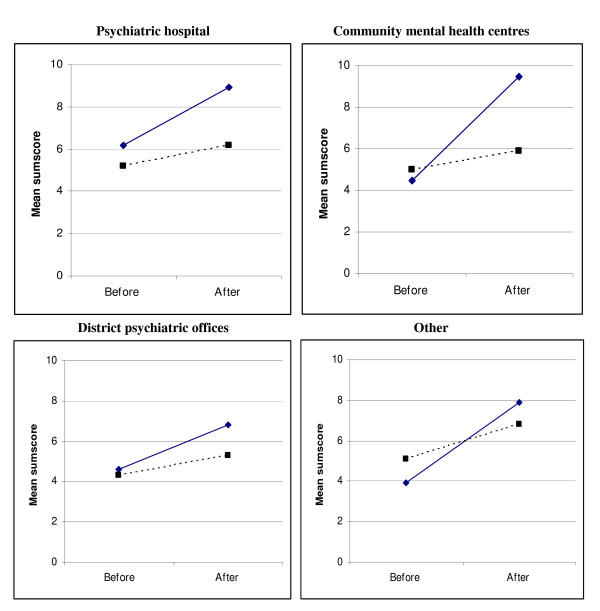
Mean sumscore for the intervention group (filled lines) and control group (dotted lines) before and after the education program by work setting.

In the second analysis, the mean sumscore of those belonging to different working places (hospital, community mental health centres, district psychiatrists and other) were examined (Figure 4). Those employed in hospitals (equally distributed in both groups) had significantly greater accuracy before education in the intervention group, while those working at the CMHC (equally distributed in both groups) showed the greatest improvement in diagnostic accuracy after participation in the intervention group.

## Discussion

There was a significant improvement in diagnostic skills in both the intervention group and the control group. However, the intervention group improved significantly more than did the control group. The pre intervention test in the present study largely confirmed the previously noted diagnostic biases which were described in a previous study [10]. The bias was partly corrected in the intervention group but not to the same degree in the control group. When analyzing both groups together, among the background factors only the current working place impacted the outcome of the intervention.

### Change of the bias compared with the previous study

When comparing the results in the case of schizoaffective disorder to the results from the study carried out two years previously [10], it seems that the above mentioned bias in diagnostic practice as evinced by these Northern Russian psychiatrists, had diminished before introduction of the specific diagnostic practice training in this study. Approximately 40% of participants in both groups made correct diagnoses compared to 13% in the previous study in 2004. This improvement over time without special education can be explained by several factors. The Russian psychiatrists have become more familiar with the current classification system, and the definition of schizoaffective disorders has probably become more accepted. The collaboration between the Russian and Norwegian psychiatrists has increased over time, with regular joint conferences and courses – something that has likely provided another perspective upon diagnostic practice. However, it is interesting to notice that other biases mentioned in the previous study (over-diagnosing of schizophrenia and schizophrenia-like diagnoses in the case of acute polymorphic psychosis and the use of somatoform disorders in the case of agoraphobia) has not changed during the two year interval between the studies.

### Change of the bias after education

The general improvement across diagnoses was more pronounced in the intervention than in the control group. The previous diagnostic bias that was observed before education in both groups was significantly decreased in the intervention group. Actually, after education there was indication on the bias only in one case in the intervention group – Acute polymorphic psychotic disorder without symptoms of schizophrenia. The number of those who felt this was a case of paranoid schizophrenia did not change after the training, indicating that the well established Russian schizophrenia-concept [17] was less affected by the training than we had expected. Alternately, the vignette may not be as specific as the ICD-10 experts had thought.

The phenomenon "agoraphobia" was relatively unknown and less used among psychiatrists working in the Archangelsk County. We have previously put forward the notion that the Russian clinicians see the somatic symptoms as more central and thus view the case as a somatoform disorder. Somatoform disorders have been more traditional diagnoses in Russia [10]. The general level of distress in Russia is probably higher than in Western countries. The level of general psychiatric morbidity has increased – especially with the levels of neurotic, stress-related and somatoform disorders increasing by 35% in recent years [18]. Presently 54% of the patients at general hospitals in Russia are considered to have a psychosomatic disorder, 27% of these with somatoform disorders [19]. Another possible explanation might be that a clinician, working in a psychiatric hospital in Russia and responsible for 30–40 patients, must be selective and trained to focus on the symptoms that might indicate organic disorders or a deterioration in the general medical condition of a patient [10]. Despite of these objective and subjective factors, the bias was significantly reduced after the structured diagnostic training (from 4.4% correct answers to 60%). A relatively high number of psychiatrists thought the vignette suggested a personality disorder (from 0% to 17.8%) after training. This can reflect a change in diagnostic thinking, from a more somatic or somatoform orientation to one of a more psychological focus.

It was somewhat surprising that the diagnostic bias was not affected by the general educational program that the control group took part in. The information about all psychiatric disorder groups, new approaches in diagnostic practice and ICD-10 had been one of the largest components of the 2-month educational program. A general educational approach was obviously not enough to change the diagnostic traditions and clinical thinking. Only a more careful and detailed training in the use of the ICD-10 in conjunction with the use of clinical vignettes, led to a marked change in the diagnostic assessments. Subjective factors such as personal/professional experience of the teacher/trainer could have had a greater impact in the less structured educational context. In the context of a diagnostic training, the use of standardized clinical vignettes, written cases, may minimize the influence of the more subjective factors. Thus, we believe that the coding of diagnoses is a special skill that requires specific training.

### Influence of the work place

It was not surprising that the psychiatrists working at the mental hospital were initially the better diagnosticians. They are considered the most qualified specialists in the county as they are working at the hub of the region's mental health system. The regional mental health system is still hospital-oriented and little decentralized [20]. Thus, most of the patients with severe mental disorders are hospitalized several times during their lives. However, most of the patients with less severe disorders are supposed to be treated by the local psychiatrists and at the community mental health centers. This results in the Russian hospital-based psychiatrists having considerably less experience with neurotic and stress-related disorders than their colleagues working at a CMHC. In addition, there is no rotation between outpatient and inpatient departments in Archangelsk County.

### Strengths and limitations of the study

The recruiting of the participants occurred in the context of the mandatory continuing education for Russian psychiatrists. Participants were from all levels of the Russian mental health system. Thus, the participants were not self selected by their motivation to improve their diagnostic skills. The control and intervention groups differed little. Their assignment to control or intervention occurred only by their chance enrollment in the obligatory education groups which alternately became the control and intervention groups in this study. All participants were available for post testing.

However, the study had some limitations. First, all participants represented the mental health services in Archangelsk County and most of them were educated and trained in psychiatry at the Medical University in Archangelsk. For this reason the generalization of the results to all Russian psychiatrists cannot be made. The second limitation relates to the possibility of a learning effect from the pre test. The same cases were used again at the post test. Thus, improvement could be partly explained by the effect of repetition. However, both groups evaluated the same 12 case vignettes that addressed the spectrum of psychiatric diagnoses. Further, the time between the two test points was 7 weeks. As the diagnostic skills were tested in the artificial situation of the educational program, we can not conclude that the focused intervention actually changed the clinicians' everyday diagnostic practices.

The present study is primarily aimed at studying the inter-rated reliability of diagnoses. The question about validity of the diagnoses is not covered. The possibility that some of the Russian diagnostic concepts may be valid needs to be further investigated.

## Conclusion

Establishing an internationally accepted diagnosis seems to be a special skill that requires specific training and needs to be an explicit part of the professional educational activities of psychiatrists. It does not appear that that skill is honed without specific training.

The issue of national diagnostic biases should be taken into account in comparative cross-cultural studies of almost any character. Such biases are complicated and need further consideration in future research. Future research should also address the question as to whether the observed improvement in diagnostic skills after specific training actually leads to changes in routine diagnostic practice.

## Competing interests

The author(s) declare that they have no competing interests.

## Authors' contributions

GR was responsible for designing the study, conducting the literature review and writing of the manuscript; participated in data analysis. AP was responsible for data collection and participated in data analysis. EF participated in data collection and data analysis. RO was responsible for designing the study, data analysis and drafting the manuscript. All authors have read the manuscript and approved it for publication.

## Pre-publication history

The pre-publication history for this paper can be accessed here:


